# Insulin resistance, adiponectin and adverse outcomes following elective cardiac surgery: a prospective follow-up study

**DOI:** 10.1186/1749-8090-5-129

**Published:** 2010-12-14

**Authors:** Martin M Mikkelsen, Troels K Hansen, Jakob Gjedsted, Niels H Andersen, Thomas D Christensen, Vibeke E Hjortdal, Søren P Johnsen

**Affiliations:** 1 Department of Cardiothoracic and Vascular Surgery T & Institute of Clinical Medicine, Aarhus University Hospital, Skejby, Brendstrupgaardsvej 100, 8200 Aarhus N, Denmark; 2 Department of Clinical Epidemiology, Aarhus University Hospital, Olof Palmes Allé, 8200 Aarhus N, Denmark; 3 Department of Endocrinology and Medical Research Laboratory, Aarhus University Hospital, Nørrebrogade, 8000 Aarhus C, Denmark; 4 Department of Cardiology, Aarhus University Hospital, Skejby, Brendstrupgaardsvej 100, 8200 Aarhus N, Denmark

## Abstract

**Background:**

Insulin resistance and adiponectin are markers of cardio-metabolic disease and associated with adverse cardiovascular outcomes. The present study examined whether preoperative insulin resistance or adiponectin were associated with short- and long-term adverse outcomes in non-diabetic patients undergoing elective cardiac surgery.

**Methods:**

In a prospective study, we assessed insulin resistance and adiponectin levels from preoperative fasting blood samples in 836 patients undergoing cardiac surgery. Population-based medical registries were used for postoperative follow-up. Outcomes included all-cause death, myocardial infarction or percutaneous coronary intervention, stroke, re-exploration, renal failure, and infections. The ability of insulin resistance and adiponectin to predict clinical adverse outcomes was examined using receiver operating characteristics.

**Results:**

Neither insulin resistance nor adiponectin were statistically significantly associated with 30-day mortality, but adiponectin was associated with an increased 31-365-day mortality (adjusted odds ratio 2.9 [95% confidence interval 1.3-6.4]) comparing the upper quartile with the three lower quartiles. Insulin resistance was a poor predictor of adverse outcomes. In contrast, the predictive accuracy of adiponectin (area under curve 0.75 [95% confidence interval 0.65-0.85]) was similar to that of the EuroSCORE (area under curve 0.75 [95% confidence interval 0.67-0.83]) and a model including adiponectin and the EuroSCORE had an area under curve of 0.78 [95% confidence interval 0.68-0.88] concerning 31-365-day mortality.

**Conclusions:**

Elevated adiponectin levels, but not insulin resistance, were associated with increased mortality and appear to be a strong predictor of long-term mortality. Additional studies are warranted to further clarify the possible clinical role of adiponectin assessment in cardiac surgery.

**Trial Registration:**

The Danish Data Protection Agency; reference no. 2007-41-1514.

## Background

Insulin resistance and circulating levels of adiponectin are associated with an increased risk of cardiovascular disease, the metabolic syndrome and a subclinical inflammatory response in the vascular endothelium [1,2].

Insulin resistance is a measure of the biological efficiency of the endogenously produced insulin and is present when a higher than normal level of insulin is required in order to maintain normoglycemia. Its prevalence in the apparently healthy population is rising [3]. However, it also declines during critical illness and as a response to surgery [1]. In a recently published study in patients undergoing cardiac surgery, intraoperative insulin resistance was associated with an increased risk of short-term adverse outcomes [4]. Moreover, hyperglycemia during cardiopulmonary bypass and preoperative metabolic syndrome, in which insulin resistance plays a key role, were powerful risk factors of mortality and morbidity in patients undergoing cardiac surgery [5,6].

Adiponectin, a hormone derived from the adipose tissue, is considered an insulin sensitizer and it upholds both anti-atherogenic and anti-inflammatory effects [2,7,8]. In non-healthy individuals, high levels of adiponectin have been associated with an increased cardiovascular disease risk in patients presenting with chest pain, increased mortality in patients with chronic heart failure, and predictive of survival after peripheral artery bypass surgery [9-11].

This strongly indicates that patients with insulin resistance or elevated adiponectin levels may have certain subclinical features, such as chronic low-grade inflammation, that can increase the risk related to cardiac surgery. Further insights in the relation between metabolic risk-markers in cardiac surgery could potentially open new avenues for improving pre-, per-, and postoperative care, but could also prove useful for preoperative risk assessment.

Indeed, improvement of risk prediction in cardiac surgery has been requested, as the EuroSCORE overestimates mortality in low-risk patients [12]. We therefore face a need to address new adverse outcome markers, including preoperative insulin resistance and adiponectin which have attracted practically no attention concerning preoperative risk prediction in cardiac surgery.

Accordingly, the aim of this study was to examine whether preoperative insulin resistance or the level of circulating adiponectin were associated with either short-term adverse outcomes within 30 days or long-term adverse outcomes (31-365 days). Secondly, we aimed to assess if information on these factors may potentially be useful for risk prediction in non-diabetic patients undergoing elective cardiac surgery.

## Methods

### Design and Setting

We conducted a single-center prospective follow-up study in the Central Denmark Region, which has a mixed rural-urban population of approximately 1.2 million. From 1 April 2005 to 30 September 2007 we included patients undergoing elective cardiac surgery at the Department of Cardiothoracic and Vascular Surgery at Aarhus University Hospital, Skejby, Denmark. The study complied to the Helsinki declaration and all patients gave informed consent prior to inclusion. The study protocol was approved by the Regional Ethics Committee and the Danish Data Protection Agency (Reference no. 2007-41-1514).

### Study population

Inclusion criteria were i) age older than 18 years, ii) elective cardiac surgery (surgery performed more than two days after planning of the procedure) - including on- and off-pump coronary artery bypass grafting, valve surgery, thoracic aortic surgery, pulmonary thrombendarterectomy, grown up congenital heart disease procedures. Exclusion criteria were i) Type I and Type II diabetes mellitus, ii) fasting blood glucose value above or equal to 7.0, or iii) previous heart transplant surgery. During the study period a total of 2,216 patients underwent cardiac surgery at the department. Patient screening and recruitment was done by a project nurse working half-time. Approximately 50% (n = 1193) of the potential candidates for the study were therefore screened consecutively. We included 876 patients with no prior history of diabetes. A preoperative in-hospital baseline fasting blood sample identified 38 patients with increased blood glucose levels above the diabetic exclusion criteria. One patient was excluded due to failure of insulin analysis, and one patient emigrated, leaving 836 patients available for 30-day (short-term) and 31-365 days (long-term) follow-up.

### Laboratory analyses

For each participant a preoperative fasting blood sample was collected (between 6 a.m. and 11 a.m.) and analyzed at the Department of Clinical Biochemistry, Aarhus University Hospital, Skejby, Denmark, and at the Medical Research Laboratory, Aarhus University Hospital, Aarhus Sygehus, Noerrebrogade, Denmark.

The fasting blood glucose values (mmol/liter) were measured in duplicate immediately after sampling on a glucose analyzer (Beckman Instruments, Palo Alto, CA), and blood insulin values (pmol/liter) were measured using a commercial immunological kit (DAKO, Glostrup, Denmark). For insulin, the intraassay coefficient of variation (CV) was 2.1-3.7%, and the interassay CV was 3.4-4.0%. We calculated the insulin resistance using the homeostasis model assessment (HOMA), where the calculation of HOMA is based on the relationship between fasting glucose and insulin levels.

HOMA=(Glucose[mmol/liter]×Insulin[mU/liter])/22.5.

The used constant converting insulin from pmol/liter to mU/liter was 6.945. Serum adiponectin (mg/liter) was measured by an in-house time-resolved immunofluorometric assay (R&D Systems, Abingdon, United Kingdom). Intra- and interassay CV averaged less than 5 and 10%, respectively.

### Study outcomes

The study outcomes were a composite of i) all-cause mortality, myocardial infarction or percutaneous coronary intervention (PCI), and stroke, and ii) deep and superficial sternal wound infection, leg wound infection (at the site of bypass graft harvest) and septicemia (defined as a positive blood culture and/or clinical sepsis). We also examined the individual elements of the composite outcomes, the risk of renal failure (defined as more than a 100% increase of serum creatinine from baseline and/or use of dialysis), risk of surgical re-exploration, as well as the length of stay in the intensive care unit and the total length of hospital stay.

Since 1968 all Danish residents have been assigned a unique civil registration number that allows unambiguous record linkage between the Danish health databases. We used the Danish Registry of Patients and the Western Denmark Heart Registry for assessing outcomes. The Danish National Registry of Patients was established in 1977 and holds data on all hospitalizations from somatic Danish hospitals, including dates of admission and discharge, procedure(s) performed, and up to 20 discharge diagnoses coded by physicians according to the International Classification of Diseases [8^th ^revision (ICD-8) until the end of 1993, end 10^th ^revision (ICD-10) thereafter]. Since 1995 discharges from emergency rooms and outpatient clinics have also been registered in this registry. The Western Denmark Heart Registry, established in 1999, is a regional clinical register including detailed patient baseline characteristics, data for all cardiac procedures performed, and per- and postoperative outcomes.

### Covariates

Baseline characteristics and in-hospital peroperative data were collected from a preoperative interview, patient medical records, the Western Denmark Heart Registry, the Prescription Database of Central Denmark Region, and the Danish National Registry of Patients. For each patient a case-report-form was used.

Baseline data included age, sex, smoking habits, body mass index, hypertension (defined as systolic pressure 140 mmHg or greater and/or diastolic pressure 90 mmHg or greater), prior ischemic peripheral, cerebro-, or cardiovascular disease, history of arrhythmias, diabetes and dyslipidemia, cardiac ejection fraction, EuroSCORE, Charlson Comorbidity Index, glomerular filtration rate as estimated by the Cockcroft Gault formula (eGFR), serum levels of creatinine, electrolytes, albumin, fructosamine, white and red blood cell counts, platelets and the urine albumin creatinine ratio.

The Charlson Comorbidity Index classifies comorbidity and in longitudinal studies it predicts both early and late mortality [13]. The index was constructed by combining data from the case-report-form with data from the National Registry of Patients, and for analyses, we categorized the index score into three levels of comorbidity: 0 ("low"), 1-2 ("medium"), and >2 ("high").

Data from the Western Denmark Heart Registry on the peroperative covariates included type of operation, cardiopulmonary bypass time and aortic cross-clamp time.

From a regional prescription database, we obtained data regarding the use of medication up to 180 days preoperatively and 1 year postoperatively. The database contains data on all redeemed prescriptions at all pharmacies in the region since 1998. The main variables are the unique civil registration number, name and drug code, package identifier (enabling identification of brand, quantity and formulation of the drug), and dates of refill.

### Statistical analyses

Baseline and procedural characteristics are presented as medians with interquartile ranges or 95% confidence intervals (95% CI) and categorical data as counts and frequencies. HOMA and adiponectin were logarithmically transformed prior to correlation with baseline and procedural characteristics. Both baseline and procedural variables were also compared across quartiles of adiponectin and HOMA using the Chi^2 ^or Kruskal-Wallis test (data not shown). Based on the quartiles of HOMA and adiponectin respectively, we divided patients into two groups. The reference groups consisted of patients with levels in the three lower quartiles (the adiponectin quartiles with the observed lowest risk) and they were compared with the upper quartiles of HOMA and adiponectin respectively.

Data on the length of intensive care unit and hospital stay were analyzed on a logarithmic scale using linear regression analyses. Thereafter, we transformed the regression estimate and estimated the absolute difference in median length of stay between groups at different levels of the EuroSCORE. The standard error was calculated using the delta method. For both short- and long-term follow-up we constructed cumulative mortality curves.

The associations between HOMA and adiponectin groups with both short- and long-term outcomes (individuals and composites) were examined using multivariate logistic regression analyses, and the associations with long-term outcomes were also examined using multivariate Cox proportional hazard analyses (for all-cause death and the composite of all-cause death, stroke and myocardial infarction/PCI) or competing risk regressions (for stroke, myocardial infarction/PCI, and infections). In the competing risk regression models, all-cause death was considered as the potential competing failure event impeding the non-fatal outcomes of interest. Using the change-in-estimate method, we examined if adjustment for possible baseline confounding factors and postoperative time-dependent use of prescribed cardiovascular drugs had impact on the risk-estimates. As there was no substantial difference between estimates from the logistic regressions and Cox or competing risk regressions, results are presented as odds ratios derived from the logistic regressions. Discrimination analyses and construction of receiver operating characteristic curves of both the uni- and multivariate models were performed to assess the predictive values of HOMA and adiponectin alone and in combination with the EuroSCORE. Hosmer-Lemeshow test was used for calibration analyses. Furthermore, we also included HOMA and adiponectin as continuous variables in an additional spline regression analysis in order to identify any non-linear patterns. A two-tailed *p*-value less than 0.05 was considered statistically significant. Analyses were performed using the Stata^® ^11.0 package (StataCorp LP, Texas, US).

## Results

### Study cohort and surgical characteristics

The overall study baseline patient characteristics and correlations with HOMA and adiponectin are shown in Table 1. For insulin resistance the upper quartile was HOMA index levels above 2.6, and for adiponectin the upper quartile was adiponectin values above 11.7 mg/liter. HOMA correlated positively with male gender, body mass index, former myocardial infarction, eGFR, glucose and insulin as well as the use of beta blockers, statins and antiplatelets. HOMA was inversely correlated with adiponectin, the EuroSCORE, microalbuminuria, type of procedure performed and cross-clamp time, but showed no correlation with age (Table 1). Adiponectin correlated positively with age, logistic EuroSCORE, urine albumin creatinine ratio, level of fructosamine, time on extra corporal circulation as well as aortic cross clamp time, and inversely with male gender, body mass index, former myocardial infarction, eGFR, and the levels of glucose, insulin and HOMA as well as the use of beta blockers and statins (Table 1). Moreover, patients with high HOMA levels had more solitary coronary bypass and less valve procedures performed, whereas increasing adiponectin levels were correlated with more valve procedures and less bypass procedures being performed (Table 1).

**Table 1:** Baseline and peroperative characteristics.

	Total sample	HOMA		Adiponectin	
Clinical features	N = 836	*r*	*p*-value	*r*	*p*-value
Male gender	607 (73)	0.14	< 0.01	-0.32	< 0.01
Age (years)	68 [59-75]	-0.06	0.08	0.15	< 0.01
BMI (kg/(m)^2^)	27 [24-30]	0.50	< 0.01	-0.38	< 0.01
Current smoker	147 (18)	< 0.01	0.99	-0.06	0.09
Hypertension	465 (56)	0.05	0.18	-0.02	0.50
EF < 50%	177 (21)	< 0.01	0.87	-0.02	0.48
MI	192 (23)	0.12	< 0.01	-0.15	< 0.01
Stroke	79 (9)	0.06	0.06	0.03	0.33
EuroSCORE	4.4 [2.2-7.8]	-0.15	< 0.01	0.29	< 0.01
Charlson Index		0.05	0.18	0.07	0.05
Low	285 (34)				
Medium	432 (52)				
High	119 (14)				
**Paraclinic**					
Creatinine (mmol/liter)	81 [68-98]	0.03	0.33	< 0.01	0.99
UACR (mg/mmol)	0.7 [0.1-1.8]	-0.05	0.13	0.16	< 0.01
Microalbuminuria	146 (18)	-0.08	0.02	0.20	< 0.01
eGFR (ml/minute)	81 [61-105]	0.23	< 0.01	-0.33	< 0.01
Glucose (mmol/liter)	5.4 [5.1-5.8]	0.52	< 0.01	-0.19	< 0.01
Fructosamine (μmol/liter)	230 [213-246]	0.02	0.61	0.21	< 0.01
Insulin (pmol/liter)	44 [30-71]	0.99	< 0.01	-0.42	< 0.01
HOMA	1.6 [1.0-2.6]			-0.42	< 0.01
Adiponectin (mg/liter)	8.0 [5.6-11.7]	-0.42	< 0.01		
**Medicine**					
RAS inhibitors*	297 (36)	0.08	0.02	-0.01	0.62
Beta blockers	521 (62)	0.14	< 0.01	-0.22	< 0.01
Statins	526 (63)	0.16	< 0.01	-0.23	< 0.01
Antiplatelets	337 (40)	0.08	0.02	-0.06	0.07
**Procedure**					
Bypass alone	326 (39)	0.16	< 0.01	-0.34	< 0.01
Valve alone	258 (31)	-0.12	< 0.01	0.22	< 0.01
Bypass & Valve	131 (16)	0.01	0.81	0.08	0.02
Others	121 (14)	-0.07	0.03	0.10	< 0.01
**Procedure related**					
ECC (minutes)	91 [68-124]	-0.04	0.19	0.14	< 0.01
CCT (minutes)	57 [40-79]	-0.08	0.01	0.20	< 0.01

Data are presented as medians [interquartile range] or absolute numbers (%)

*r *is the correlation coefficient

* Includes angiotensin-converting enzyme inhibitors and angiotensin-II receptor antagonists

AF - Atrial fibrillation or flutter; BMI - Body mass index; CCT - Cross clamp time; ECC - Extra corporal circulation; eGFR - Estimated glomerular filtration rate; EF - Ejection fraction; HOMA - Homeostasis model assessment; Kg - Kilogram; M - Meter; MI - Myocardial infarction; UACR - Urinary albumin creatinine ratio; RAS - Renin angiotensin system

### Length of stay

There was no difference between the upper quartile and the three lower quartiles of HOMA regarding median length of stay in the intensive care unit (difference: 0.02 days [95% CI -0.08-0.12]) or total hospital stay (difference: 0.20 days [95% CI -0.21-0.61]). Patients in the upper adiponectin quartile stayed 0.15 (95% CI 0.04-0.26) days longer in the intensive care unit, and had a 0.73 (95% CI 0.27-1.19) days prolonged total hospital stay as compared to the lower adiponectin quartiles and adjusted for the logistic EuroSCORE.

### Insulin resistance and postoperative adverse outcomes

**Figure 1:**
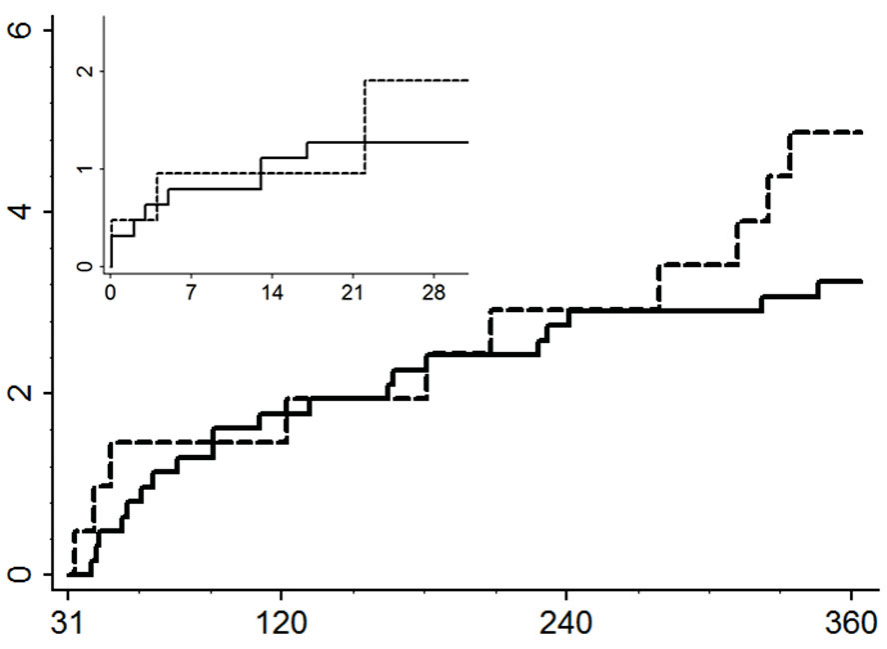
**Cumulative mortality considering HOMA quartiles**. Large graph shows the cumulative mortality from day 31 until 365 (Log rank *p *> 0.05). Small graph shows the cumulative mortality from day 0 until 30 (Log rank *p >*0.05). x-axes - Days after surgery; y-axes - Cumulative mortality (%); Dashed lines - Insulin resistance quartile 4; Solid lines - Insulin resistance quartiles 1-3; HOMA - Homeostasis model assessment

The associations between HOMA quartiles and study outcomes at both short- and long-term follow-up are displayed in Table 2. Increased HOMA values were not statistically significantly associated with postoperative mortality when compared to the lower three quartiles (30-day adjusted OR 1.7 [95% CI 0.5-5.7] and 31-365-days adjusted OR 1.7 [95% CI 0.7-3.3]) (Figure 1). For early postoperative infections, the odds ratio was 1.5, but did not reach statistical significance. Moreover, the upper HOMA quartile was also not associated with other individual or combined outcomes. Similarly, comparing groups above and below the median HOMA value showed statistically insignificant associations between HOMA and outcomes. Furthermore, analyzing HOMA as a continuous spline function revealed no specific threshold values in the association with all-cause death.

**Table 2:** Short- and long-term odds ratios considering insulin resistance.

	HOMA quartiles		Short-term follow-up			
	
	I - III	IV	Crude		Adjusted*	
	n = 627	n = 209	OR	95% CI	OR	95% CI
**Death**	8 (1.3)	4 (1.9)	1.5	1.0-9.6	1.7	0.5-5.7
**MI/PCI**	15 (3.4)	5 (3.4)	1.0	0.5-2.8	1.0	0.4-2.8
**Stroke**	23 (3.7)	8 (3.8)	1.0	0.5-2.4	1.1	0.5-2.5
**Renal failure**^†^	39 (6.2)	16 (7.7)	1.2	0.7-2.3	1.4	0.7-2.7
**Re-exploration**	54 (8.6)	22 (10.5)	1.2	0.7-2.1	1.3	0.8-2.2
**Infections**	27 (4.3)	13 (6.2)	1.5	0.7-2.9	1.5	0.8-3.0
**CVD composite**	44 (7.0)	16 (7.7)	1.1	0.6-2.0	1.1	0.6-2.1
	**HOMA quartiles**		**Long-term follow-up**			
	
	**I - III**	**IV**	**Crude**		**Adjusted**^‡^	
	**n = 619**	**n = 205**	**OR**	**95% CI**	**OR**	**95% CI**

**Death**	20 (3.2)	10 (4.9)	1.5	0.7-3.3	1.7	0.7-3.8
**MI/PCI**	18 (2.9)	4 (2.0)	0.7	0.2-2.0	0.6	0.2-1.8
**Stroke**	12 (1.9)	1 (0.5)	0.2	0.1-1.9	0.3	0.1-2.0
**Infections**	20 (3.2)	8 (3.9)	1.2	0.5-2.8	1.2	0.5-2.9
**CVD composite**	45 (7.3)	14 (6.8)	0.9	0.5-1.7	0.9	0.5-1.7

* Adjusted for the logistic EuroSCORE

^† ^Adjusted for the logistic EuroSCORE and estimated glomerular filtration rate

^‡ ^Adjusted for the logistic EuroSCORE, Charlson Comorbidity Index and type of surgery

Short-term is defined as 30-day follow-up

Long-term is defined as follow-up from day 31 until 365

CI - Confidence interval; CVD - Cardiovascular disease; HOMA - Homeostasis model assessment; MI - Myocardial infarction; OR; - Odds ratio; PCI - Percutaneous coronary intervention

### Adiponectin and postoperative adverse outcomes

**Figure 2:**
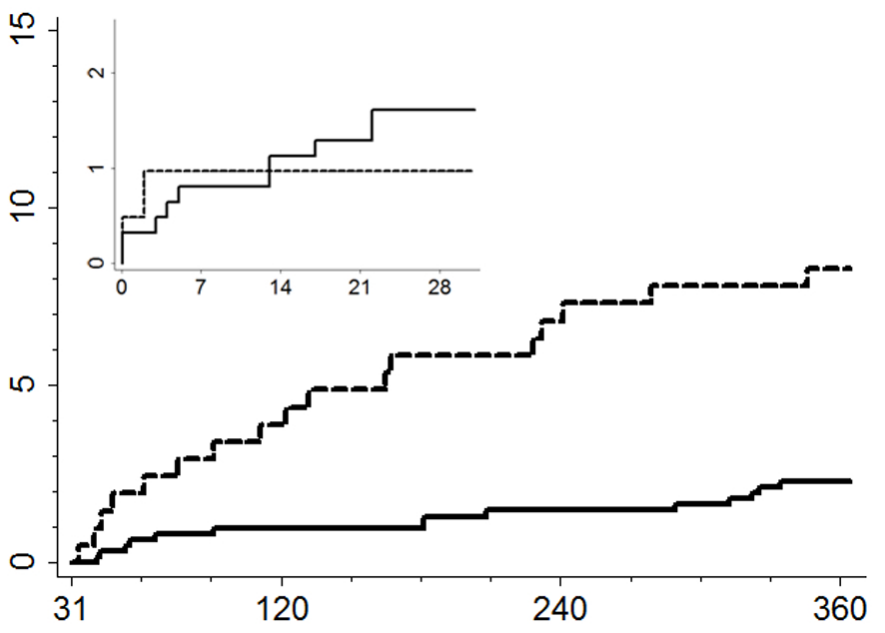
**Cumulative mortality considering adiponectin quartiles**. Large graph shows the cumulative mortality from day 31 until 365 (Log rank *p *< 0.05). Small graph shows the cumulative mortality from day 0 until 30 (Log rank *p >*0.05). x-axes: Days after surgery. y-axes: Cumulative mortality (%). Dashed lines: Adiponectin quartile 4. Solid lines: Adiponectin quartiles 1-3.

As displayed in Table 3 adiponectin was not associated with any of the short-term postoperative outcomes, except from renal failure (adjusted OR 1.8 [95% CI 1.0-3.3]. In contrast, high levels of circulating adiponectin were positively associated with all-cause death in the 31-365 days time window (adjusted OR of 2.9 [95% CI 1.3-6.4]) for patients in the upper quartile compared with patients in the lower three quartiles (Figure 2). The increased risk of the combined cardiovascular outcome in the highest adiponectin quartile (adjusted OR 1.7 [95% CI 0.9-3.1]) was primarily driven by all-cause mortality, as there were no strong associations between adiponectin and myocardial infarction/PCI or stroke. Comparing groups above and below the median adiponectin (data not shown) indicated an even higher mortality risk (adjusted OR 4.4 [95% CI 1.6-12.1]). Otherwise, the median cut-off showed no substantially different trends. Considered as a continuous variable, each 1 mg/liter increase in adiponectin was associated with a 1.12 [95% CI 1.08-1.16] increased adjusted OR for all-cause death. In the spline regression model we could not determine any specific cut-off level for adiponectin.

**Table 3:** Short- and long-term odds ratios considering adiponectin.

	Adiponectin quartiles		Short-term follow-up			
	
	I - III	IV	Crude		Adjusted*	
	n = 627	n = 209	OR	95% CI	OR	95% CI
**Death**	10 (1.6)	2 (1.0)	0.6	0.4-5.7	0.4	0.1-2.0
**MI/PCI**	15 (2.4)	5 (2.4)	1.0	0.4-2.8	1.0	0.3-2.7
**Stroke**	20 (3.2)	11 (5.3)	1.7	0.8-3.6	1.5	0.7-3.3
**Renal failure**	33 (5.3)	22 (10.5)	2.1	1.2-3.7	1.4	0.7-2.7
**Re-exploration**	54 (8.6)	22 (10.5)	1.2	0.7-2.1	0.9	0.6-1.9
**Infections**	29 (4.6)	11 (5.3)	1.1	0.6-2.3	1.0	0.5-2.1
**CVD composite**	43 (6.9)	17 (8.1)	1.2	0.7-2.2	1.0	0.6-1.9
	**Adiponectin quartiles**		**Long-term follow-up**			
	
	**I - III**	**IV**	**Crude**		**Adjusted**^‡^	
	**n = 617**	**n = 207**	**OR**	**95% CI**	**OR**	**95% CI**

**Death**	13 (2.1)	17 (8.2)	4.2	2.0-8.7	2.9	1.3-6.4
**MI/PCI**	18 (2.9)	4 (1.9)	0.7	0.2-2.0	0.7	0.2-2.1
**Stroke**	8 (1.3)	5 (2.4)	1.9	0.6-5.8	1.4	0.4-4.5
**Infections**	18 (2.9)	10 (4.8)	1.7	0.8-3.7	1.1	0.5-2.6
**CVD composite**	36 (5.8)	23 (11.1)	2.0	1.2-3.5	1.7	0.9-3.1

* Adjusted for the logistic EuroSCORE

^† ^Adjusted for the logistic EuroSCORE and estimated glomerular filtration rate

^‡ ^Adjusted for the logistic EuroSCORE, Charlson Comorbidity Index and type of surgery

Short-term is defined as 30-day follow-up

Long-term is defined as follow-up from day 31 until 365

CI - Confidence interval; CVD - Cardiovascular disease; MI - Myocardial infarction; OR - Odds ratio; PCI - Percutaneous coronary intervention

### Predictive values of HOMA, adiponectin and the EuroSCORE

The areas under the receiver operating characteristic curves (AUC) concerning mortality are shown in Table 4. The AUC was 0.84 [95% CI 0.75-0.93] for the logistic EuroSCORE regarding short-term all-cause death and 0.75 [95% CI 0.67-0.83] for long-term all-cause death. HOMA did not predict mortality. In contrast, the AUC for adiponectin was 0.75 [95% CI 0.65-0.85] regarding long-term mortality and in a model including both the EuroSCORE and adiponectin the AUC reached 0.78 [95% CI 0.68-0.88]. In a model with only HOMA and adiponectin a similar AUC was achieved, and when the EuroSCORE was then added, the AUC increased up to 0.81 [95% CI 0.73-0.89]. Lastly, adding the Charlson Comorbidity Index to the model further increased the AUC to 0.86 [95% CI 0.81-0.92]. There were no interactions between sex and insulin resistance or adiponectin with regard to the risk of any postoperative outcomes. Hosmer-Lemeshow tests showed acceptable model fit of the logistic regressions.

**Table 4:** Areas under receiver operating curves characteristics on all-cause death.

	Short-term follow-up		Long-term follow-up	
	AUC	95% CI	AUC	95% CI
Logistic EuroSCORE	0.84	0.75-0.93	0.75	0.67-0.83
HOMA continuous	0.55	0.36-0.75	0.47	0.34-0.60
HOMA quartiles	0.54	0.40-0.68	0.54	0.46-0.63
ADPN continuous	0.53	0.38-0.68	0.75	0.65-0.85
ADPN quartiles	0.54	0.43-0.65	0.66	0.57-0.76
Logistic EuroSCORE + HOMA continuous	0.84	0.76-0.92	0.77	0.70-0.84
Logistic EuroSCORE + HOMA quartiles	0.77	0.65-0.90	0.76	0.69-0.82
Logistic EuroSCORE + ADPN continuous	0.82	0.68-0.95	0.78	0.68-0.88
Logistic EuroSCORE + ADPN quartiles	0.83	0.70-0.96	0.76	0.68-0.85
HOMA and ADPN continuous			0.77	0.68-0.86
Logistic EuroSCORE + HOMA and ADPN continuous			0.81	0.73-0.89
Logistic EuroSCORE + HOMA and ADPN continuous + CCI			0.86	0.81-0.92

Short-term is defined as 30-day follow-up

Long-term is defined as follow-up from day 31 until 365

ADPN - Adiponectin; AUC - Area under curve; CI - Confidence interval; CCI - Charlson Comorbidity Index; HOMA - Homeostasis model assessment

## Discussion

In the present study, high levels of adiponectin were associated with an increased 31-365-day mortality following elective cardiac surgery. In addition, adiponectin had a predictive value corresponding to that of the EuroSCORE, whereas insulin resistance alone did not contribute with any important prognostic information on mortality.

The association between preoperative insulin resistance and short-term mortality (1.7-fold increased risk) did not reach statistical significance, but seems clinically interesting since high HOMA indices may help identify a subgroup of non-diabetic patients at higher risk - and with a possible pre- and intraoperative medical intervention available (i.e. insulin sensitizers and insulin). A recent study showed an approximately 2-fold increased risk of mortality and major adverse outcomes in patients with intraoperatively decreased insulin sensitivity [4]. A low-grade inflammation associated with insulin resistance might be accentuated during surgery, and in particular patients undergoing cardiac surgery experience aggravated inflammation and insulin resistance - which participates in a worsening of endothelial dysfunction, glycemic control, and increase risk of postoperative adverse outcomes [14-16]. Moreover, per- and postoperative aggravated insulin resistance and hyperglycemia are apparently important factors in studies documenting the effect of postoperative tight glycemic control with insulin therapy on morbidity and mortality [17,18]. However, not all studies support the notion that tight intraoperative glycemic control with insulin therapy reduces adverse outcomes following cardiac surgery [19]. The present result showed poor predictive values of preoperatively measured insulin resistance alone and therefore does not support the use of routine preoperative assessment of insulin resistance in cardiac surgery.

The association between adiponectin and all-cause death found in our study is in accordance with the results reported by Kistorp et al, who found a high adiponectin level to predict mortality in patients with congestive heart failure [10]. Moreover, the "AtheroGene study", including 1890 patients with coronary artery disease, found a positive correlation between adiponectin levels and the risk of a new cardiovascular event (HR 1.17 for each increase in adiponectin quartile) [20]. In addition, another study on adiponectin in patients with coronary artery disease indicated that high adiponectin levels was associated with an increased risk of cardiovascular death, but when controlled for potential confounding the association did not remain statistically significant [21]. However, in 2006 results from a metaanalysis indicated that low adiponectin levels were associated with a higher risk of cardiovascular disease [22]. A bidirectional association between adiponectin and cardiovascular disease influenced by the constellation of existing comorbidity appears plausible, but the role of adiponectin as a risk factor or independent prognostic marker in different constellations of comorbidities remains contracdictious and sparsely understood [21,23,24].

Preoperative assessment of adiponectin was not associated with short-term risk. However, high adiponectin levels in the present population identified patients with increased cardiovascular risk on the long term, corresponding to what was achieved by the multifactorial risk stratification contained in the EuroSCORE.

The EuroSCORE is a sensitive predictor of 30-day postoperative mortality, but it has been shown to overestimate mortality in low-risk patients and to underestimate mortality in high-risk patients [12]. Therefore, it is important to improve risk prediction both with and beyond the EuroSCORE (and other alternative risk assessment tools) by investigating the predictive ability of new potential markers of risk. In the present study, neither the HOMA index nor adiponectin levels assessed in a preoperative fasting blood sample contributed with better risk prediction regarding the adverse 30-day postoperative outcomes than the EuroSCORE itself. Nevertheless, our results suggest that preoperative assessment of especially adiponectin levels may contribute with additional risk stratification and especially help identify patients with increased long-term risk. However, since elective cardiac surgery in general is considered to be safe with a low mortality, a larger number of patients and morbid events may however be required to demonstrate improved accuracy of the logistic EuroSCORE from assessment of either insulin resistance or adiponectin.

### Limitations and strengths

The study design does not allow us to infer causality between the insulin resistance, adiponectin and postoperative outcomes. Even so, we studied a well-defined cohort that was representative of the patient population undergoing cardiac surgery at our department. We had a practically complete follow-up on all included patients, since our design relied on population-based registries with complete coverage. Recruitment of participants was prospective and independent of exposure levels. Besides that, the levels of insulin resistance and adiponectin were not known to the surgeons and physicians treating the patients and therefore the risk of information bias was minimal. When considering registry data validity, the predictive value have previously been reported to be high (approximately 80-99%) for several of the outcomes in our study including myocardial infarction and stroke [25,26]. Any misclassification would in any case most likely be independent of the level of insulin resistance and adiponectin and would bias the findings toward the null hypothesis. Although insulin is excreted in a pulsatile fashion, and the average of three independent samples would be a more precise estimate of the true plasma insulin value, the use of only one sample is acceptable and yields similar results compared to three samples in large datasets [27].

## Conclusions

In conclusion, high levels of preoperative insulin resistance or adiponectin are not associated with increased 30-day mortality, but a high level of adiponectin implies an increased 31-365-day mortality, and slightly prolonged length of intensive care unit and total hospital stay. Owing to our results on prognostic values, we suggest additional studies to further clarify the potentially important role of preoperative insulin resistance and in particular adiponectin in preoperative risk assessment in cardiac surgery.

## Competing interests

The authors declare that they have no competing interests.

## Authors' contributions

MMM: principal investigator. All authors: study design. MMM, TKH, TDC, VH, SPJ: data aquisition. MMM and SPJ: data analyses. MMM: article writing. MMM, TKH, JG, NHA, TDC, VH, SPJ: critical reviews of article drafts and approval of the final version to be published.

## References

[B1] Bagry HS Raghavendran S Carli F Metabolic syndrome and insulin resistance: perioperative considerations Anesthesiology 2008 108 506 523 10.1097/ALN.0b013e3181649314 18292688

[B2] Shibata R Ouchi N Murohara T Adiponectin and cardiovascular disease Circ J 2009 73 608 614 10.1253/circj.CJ-09-0057 19261992

[B3] Ioannou GN Bryson CL Boyko EJ Prevalence and trends of insulin resistance, impaired fasting glucose, and diabetes J Diabetes Complications 2007 21 363 370 10.1016/j.jdiacomp.2006.07.005 17967708

[B4] Sato H Carvalho G Sato T Lattermann R Matsukawa T Schricker T The Association of Preoperative Glycemic Control, Intraoperative Insulin Sensitivity, and Outcomes after Cardiac Surgery J Clin Endocrinol Metab 2010 95 9 4338 44 Epub 2010 Jul 14 10.1210/jc.2010-0135 20631016

[B5] Echahidi N Pibarot P Despres JP Daigle JM Mohty D Voisine P Baillot R Mathieu P Metabolic syndrome increases operative mortality in patients undergoing coronary artery bypass grafting surgery J Am Coll Cardiol 2007 50 843 851 10.1016/j.jacc.2007.04.075 17719470

[B6] Doenst T Wijeysundera D Karkouti K Zechner C Maganti M Rao V Borger MA Hyperglycemia during cardiopulmonary bypass is an independent risk factor for mortality in patients undergoing cardiac surgery J Thorac Cardiovasc Surg 2005 130 1144 10.1016/j.jtcvs.2005.05.049 16214532

[B7] Matsuda M Shimomura I Sata M Arita Y Nishida M Maeda N Kumada M Okamoto Y Nagaretani H Nishizawa H Kishida K Komuro R Ouchi N Kihara S Nagai R Funahashi T Matsuzawa Y Role of adiponectin in preventing vascular stenosis. The missing link of adipo-vascular axis J Biol Chem 2002 277 37487 37491 10.1074/jbc.M206083200 12138120

[B8] Weyer C Funahashi T Tanaka S Hotta K Matsuzawa Y Pratley RE Tataranni PA Hypoadiponectinemia in obesity and type 2 diabetes: close association with insulin resistance and hyperinsulinemia J Clin Endocrinol Metab 2001 86 1930 1935 10.1210/jc.86.5.1930 11344187

[B9] Cavusoglu E Ruwende C Chopra V Yanamadala S Eng C Clark LT Pinsky DJ Marmur JD Adiponectin is an independent predictor of all-cause mortality, cardiac mortality, and myocardial infarction in patients presenting with chest pain Eur Heart J 2006 27 2300 2309 10.1093/eurheartj/ehl153 16864609

[B10] Kistorp C Faber J Galatius S Gustafsson F Frystyk J Flyvbjerg A Hildebrandt P Plasma adiponectin, body mass index, and mortality in patients with chronic heart failure Circulation 2005 112 1756 1762 10.1161/CIRCULATIONAHA.104.530972 16157772

[B11] Komai H Shibata R Juri M Matsushita K Ouchi N Murohara T Plasma adiponectin as a predictive factor of survival after a bypass operation for peripheral arterial disease J Vasc Surg 2009 50 95 99 10.1016/j.jvs.2008.12.044 19563957

[B12] Choong CK Sergeant P Nashef SA Smith JA Bridgewater B The EuroSCORE risk stratification system in the current era: how accurate is it and what should be done if it is inaccurate? Eur J Cardiothorac Surg 2009 35 59 61 10.1016/j.ejcts.2008.10.009 19027318

[B13] de G Beckerman H Lankhorst GJ Bouter LM How to measure comorbidity. a critical review of available methods J Clin Epidemiol 2003 56 221 229 10.1016/S0895-4356(02)00585-1 12725876

[B14] Farrer M Fulcher G Albers CJ Neil HA Adams PC Alberti KG Patients undergoing coronary artery bypass graft surgery are at high risk of impaired glucose tolerance and diabetes mellitus during the first postoperative year Metabolism 1995 44 1016 1027 10.1016/0026-0495(95)90099-3 7637643

[B15] Knapik P Nadziakiewicz P Urbanska E Saucha W Herdynska M Zembala M Cardiopulmonary bypass increases postoperative glycemia and insulin consumption after coronary surgery Ann Thorac Surg 2009 87 1859 1865 10.1016/j.athoracsur.2009.02.066 19463610

[B16] Rapp-Kesek D Stridsberg M Andersson LG Berne C Karlsson T Insulin resistance after cardiopulmonary bypass in the elderly patient Scand Cardiovasc J 2007 41 102 108 10.1080/14017430601050355 17454835

[B17] Van den BG Wouters P Weekers F Verwaest C Bruyninckx F Schetz M Vlasselaers D Ferdinande P Lauwers P Bouillon R Intensive insulin therapy in the critically ill patients N Engl J Med 2001 345 1359 1367 10.1056/NEJMoa011300 11794168

[B18] Lazar HL Chipkin SR Fitzgerald CA Bao Y Cabral H Apstein CS Tight glycemic control in diabetic coronary artery bypass graft patients improves perioperative outcomes and decreases recurrent ischemic events Circulation 2004 109 1497 1502 10.1161/01.CIR.0000121747.71054.79 15006999

[B19] Gandhi GY Nuttall GA Abel MD Mullany CJ Schaff HV O'Brien PC Johnson MG Williams AR Cutshall SM Mundy LM Rizza RA McMahon MM Intensive intraoperative insulin therapy versus conventional glucose management during cardiac surgery: a randomized trial Ann Intern Med 2007 146 233 243 17310047 10.7326/0003-4819-146-4-200702200-00002

[B20] Schnabel R Messow CM Lubos E Espinola-Klein C Rupprecht HJ Bickel C Sinning C Tzikas S Keller T Genth-Zotz S Lackner KJ Munzel TF Blankenberg S Association of adiponectin with adverse outcome in coronary artery disease patients: results from the AtheroGene study Eur Heart J 2008 29 649 657 10.1093/eurheartj/ehn009 18263867

[B21] Maiolino G Cesari M Sticchi D Zanchetta M Pedon L Antezza K Pessina AC Rossi GP Plasma adiponectin for prediction of cardiovascular events and mortality in high-risk patients J Clin Endocrinol Metab 2008 93 3333 3340 10.1210/jc.2007-2405 18697874

[B22] Sattar N Wannamethee G Sarwar N Tchernova J Cherry L Wallace AM Danesh J Whincup PH Adiponectin and coronary heart disease: a prospective study and meta-analysis Circulation 2006 114 623 629 10.1161/CIRCULATIONAHA.106.618918 16894037

[B23] Laughlin GA Barrett-Connor E May S Langenberg C Association of adiponectin with coronary heart disease and mortality: the Rancho Bernardo study Am J Epidemiol 2007 165 164 174 10.1093/aje/kwk001 17101706 PMC2642645

[B24] Shibata R Ouchi N Murohara T Adiponectin and cardiovascular disease Circ J 2009 73 608 614 10.1253/circj.CJ-09-0057 19261992

[B25] Joensen AM Jensen MK Overvad K Dethlefsen C Schmidt E Rasmussen L Tjonneland A Johnsen S Predictive values of acute coronary syndrome discharge diagnoses differed in the Danish National Patient Registry J Clin Epidemiol 2008 66 188 194 10.1016/j.jclinepi.2008.03.005 18722087

[B26] Johnsen SP Overvad K Sorensen HT Tjonneland A Husted SE Predictive value of stroke and transient ischemic attack discharge diagnoses in The Danish National Registry of Patients J Clin Epidemiol 2002 55 602 607 10.1016/S0895-4356(02)00391-8 12063102

[B27] Wallace TM Levy JC Matthews DR Use and abuse of HOMA modeling Diabetes Care 2004 27 1487 1495 10.2337/diacare.27.6.1487 15161807

